# Asymmetrical hippocampal connectivity in mesial temporal lobe epilepsy: evidence from resting state fMRI

**DOI:** 10.1186/1471-2202-11-66

**Published:** 2010-06-02

**Authors:** Fabrício RS Pereira, Andréa Alessio, Maurício S Sercheli, Tatiane Pedro, Elizabeth Bilevicius, Jane M Rondina, Helka FB Ozelo, Gabriela Castellano, Roberto JM Covolan, Benito P Damasceno, Fernando Cendes

**Affiliations:** 1 Neuroimaging Laboratory, Department of Neurology, University of Campinas - UNICAMP, Cidade Universitária, Campinas, SP, Brazil; 2 Neurophysics Group, Gleb Wataghin Institute of Physics, P.O. Box 6165, University of Campinas - UNICAMP, Zip Code 13083-970, Campinas, SP, Brazil

## Abstract

**Background:**

Mesial temporal lobe epilepsy (MTLE), the most common type of focal epilepsy in adults, is often caused by hippocampal sclerosis (HS). Patients with HS usually present memory dysfunction, which is material-specific according to the hemisphere involved and has been correlated to the degree of HS as measured by postoperative histopathology as well as by the degree of hippocampal atrophy on magnetic resonance imaging (MRI). Verbal memory is mostly affected by left-sided HS, whereas visuo-spatial memory is more affected by right HS. Some of these impairments may be related to abnormalities of the network in which individual hippocampus takes part. Functional connectivity can play an important role to understand how the hippocampi interact with other brain areas. It can be estimated via functional Magnetic Resonance Imaging (fMRI) resting state experiments by evaluating patterns of functional networks. In this study, we investigated the functional connectivity patterns of 9 control subjects, 9 patients with right MTLE and 9 patients with left MTLE.

**Results:**

We detected differences in functional connectivity within and between hippocampi in patients with unilateral MTLE associated with ipsilateral HS by resting state fMRI. Functional connectivity resulted to be more impaired ipsilateral to the seizure focus in both patient groups when compared to control subjects. This effect was even more pronounced for the left MTLE group.

**Conclusions:**

The findings presented here suggest that left HS causes more reduction of functional connectivity than right HS in subjects with left hemisphere dominance for language.

## Background

Temporal lobe epilepsy (TLE) is one of the most frequent forms of refractory partial epilepsies. It has a variety of causes such as strokes, tumors and malformations [1]. However, the most common cause of TLE in surgical series is hippocampal sclerosis (HS), which can reliably be detected *in vivo *by MRI [2,3]. Structural damage in TLE associated with HS is a condition that characterizes mesial temporal lobe epilepsy (MTLE) [3,4]. Such damage and dysfunction frequently extend beyond the hippocampus, since individuals with refractory MTLE quite often exhibit hippocampal, parahippocampal and entorhinal cortex atrophies [5]. This structural damage is usually associated with memory impairments [6]. Patients with left MTLE have a tendency to present verbal memory deficits whereas subjects with right MTLE may exhibit deficits of non-verbal (visual) memory [7,8].

In general, MTLE seizures are generated in the hippocampus [9] and frequently propagate to other limbic structures [10]. In addition, interictal epileptiform discharges can also spread through tracts from the focus to other brain areas [11]. Observations support the hypothesis of connectivity between hippocampus and other brain structures [12], but it is not clear whether the functional and structural architectures of these interactions play similar or different roles in normal controls and patients with MTLE [13,14]. In order to investigate this issue, we sought differences in functional connectivity among brain areas that are usually affected by the MTLE syndrome.

One of the ways to define functional connectivity is in terms of temporal correlations between remote neurophysiological events [15]. These events may be, for instance, hemodynamic responses in fMRI experiments [16], simultaneously recorded spiking activity and local field potentials (LFP) [17], or metabolic measurements in PET/SPECT experiments [18].

Different neuroimaging modalities have been employed to estimate functional connectivity in epilepsy, such as repetitive transcranial magnetic stimulation (rTMS) [19], cortico-cortical evoked potentials (CCEP) [20] and EEG [21]. These techniques have the advantage that they are noninvasive, but for resting state fMRI analysis there is an additional gain: it is possible to obtain functional network information even when no specific task is performed, allowing the search for significant baseline fluctuations.

Synchronized low-frequency fluctuations of BOLD-fMRI signals have been observed between remote brain areas during resting state [22], as well as in event related [23] and blocked design paradigms [24]. This synchrony may indicate normal or pathologic patterns in a neuronal network. Therefore, measurements of functional connectivity figure importantly in neurophysiological and neuropsychological investigations, especially for elucidating mechanisms of integration and segregation of brain information. In order to explore differences of this synchrony with respect to BOLD signal changes, we applied the methodologies of functional connectivity in groups of patients with unilateral (right and left) HS and control subjects.

## Results

We compared the level of functional connectivity of left and right hippocampi by considering three groups: a control group, patients with right MTLE and patients with left MTLE.

**Figure 1:**
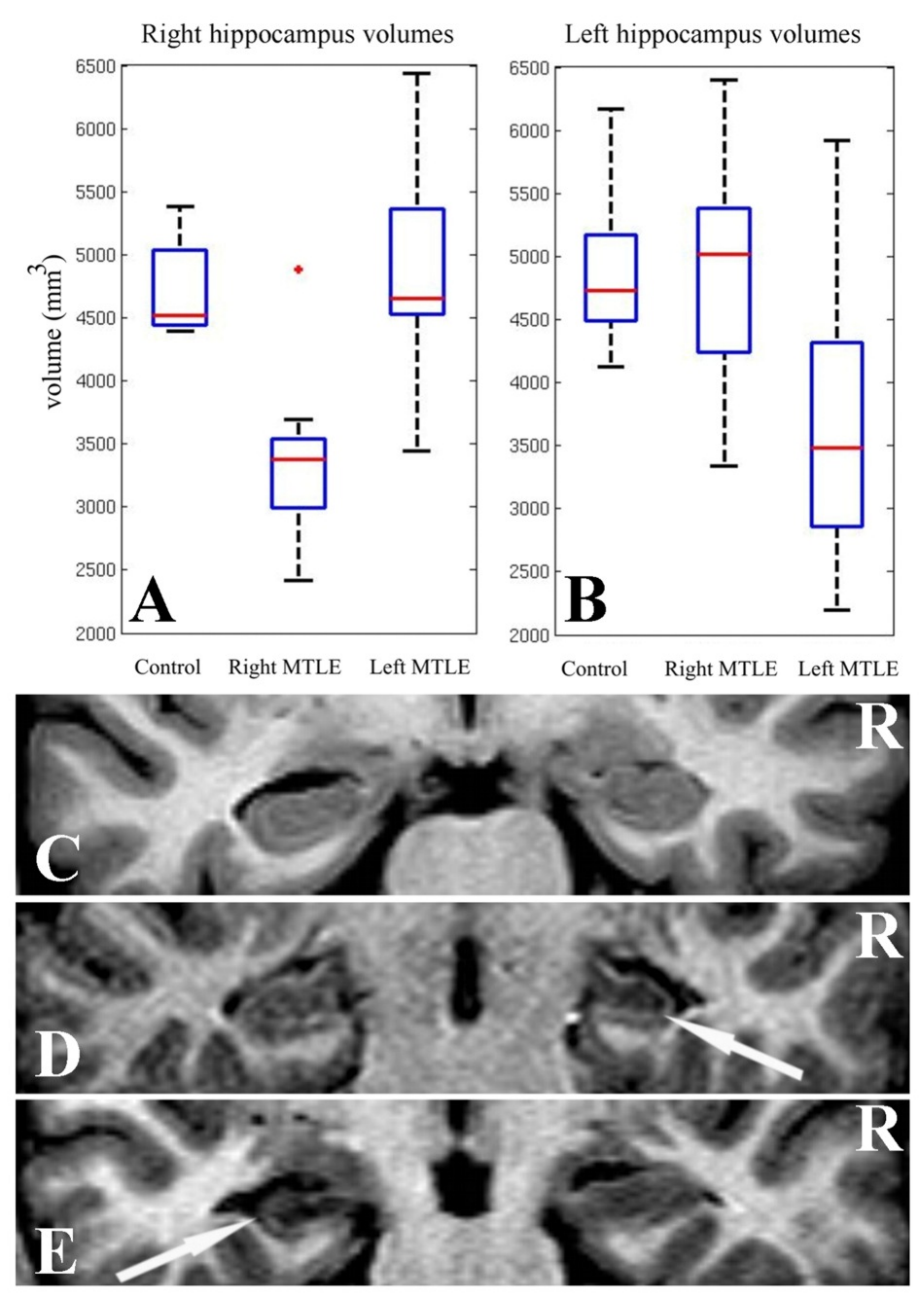
**Hippocampal manual volumetric analyses**. A) Distribution of the volume of right hippocampus across the groups: control, right MTLE and left MTLE. B) Distribution of the volume of left hippocampus across the groups: control, right MTLE and left MTLE. C) Coronal structural image of normal subject. D) Coronal structural image of patient with right MTLE: Arrow indicates the right hippocampal sclerosis. E) Coronal structural image of patient with left MTLE: Arrow indicates the left hippocampal sclerosis. Abbreviations - R: right hemisphere.

Manual volumetric analyses showed a significant atrophy of the ipsilateral hippocampus in each group of patients compared to controls (T = 5.33, p < 0.001 for right MTLE and T = 2.41, p < 0.05 for left MTLE), but no signs of atrophy on the contralateral side. These outcomes can be seen in Figure 1. In addition, the degree of atrophy of the ipsilateral hippocampus was not different between patient groups. The quantitative asymmetric index (QAI) revealed significant differences between the QAI of the control and each group of patients (T = -5.33, p < 0.001 for right MTLE and T = -3.18, p < 0.01 for left MTLE), whereas no significant differences (T = 0.85, p < 0.5) were found for QAI between patients' groups (Table 1). Figure 1 also illustrates a structural MRI of a normal subjects (Figure 1C), a patient with right MTLE (Figure 1D) associated to right HS (arrow in Figure 1D) and a patient with left MTLE (Figure 1E) associated to left HS (arrow in Figure 1E).

**Table 1:** Hippocampal volumetric data and comparison between groups

	Manual volumetric measurements								
	*Control Group*			*Right MTLE group*			*Left MTLE group*		
Subject	VLH*(mm3)*	VRH*(mm3)*	QAI	VLH*(mm3)*	VRH*(mm3)*	QAI	VLH*(mm3)*	VRH*(mm3)*	QAI
**1**	4126	4964	0.17	4437	3215	0.28	2589	4545	0.4304
**2**	4607	4398	0.05	6400	4885	0.24	5919	6440	0.0809
**3**	4852	4521	0.07	3340	2416	0.28	3902	4476	0.1282
**4**	6170	5266	0.15	5122	3689	0.28	3384	4777	0.2916
**5**	4979	4521	0.09	5014	3033	0.40	5551	5770	0.0380
**6**	4312	4438	0.03	3841	3493	0.09	3842	4653	0.1743
**7**	4545	4436	0.02	4371	3373	0.23	2194	3440	0.3622
**8**	4728	4938	0.04	5143	3408	0.34	2943	5224	0.4366
**9**	5740	5378	0.06	6113	2870	0.53	3481	4601	0.2434
**mean**	4895.4	4762.2	0.08	4864.6	3375.8	0.29	3756.1	4880.7	0.2428
**std**	662.7	381.6	0.05	992.5	681.1	0.12	1255.3	853.9	0.1481

	Ipsilateral comparisons								
	*Control vs Right MTLE*			*Control vs Left MTLE*			*Right MTLE vs Left MTLE*		
	**VLH**	**VRH**	**QAI**	**VLH**	**VRH**	**QAI**	**VLH**	**VRH**	**QAI**
									
**T-score**	0.08	5.33	-5.33	2.41	-0.38	-3.18	2.08	-4.13	0.85
**P-value**	0.94	<0.001	<0.001	<0.05	0.71	<0.01	<0.05	<0.001	0.41

	Contralateral comparisons								
	**VLH ***of Right MTLE*			**VRH ***of Right MTLE*					
	group VS			group VS					
	**VRH ***of Left MTLE group*			**VLH ***of Left MTLE group*					
									
**T-score**	-0.04			-0.80					
**P-value**	0.97			0.44					

VLH, volume of left hippocampus; VRH, volume of right hippocampus; QAI, quantitative asymmetric index.

Neuropsychological data from both patients' groups demonstrated significant differences with lower scores in left MTLE group for the following tests: WAIS-R estimated IQ (T = 3.11, p < 0.01); WMS-R General Memory (T = 3.92, p < 0.005); WMS-R Verbal Memory (T = 3.81, p < 0.005) and WMS-R Delayed Recall (T = 2.52, p < 0.05). The other neuropsychological tests (Boston Naming Test, Verbal Fluency Test, Vigilance Test and WMS-R Visual Memory) did not differ between patients' groups (Table 2).

**Table 2:** Neuropsychological data of patients

Patients	Dichotic Listening Test	WAIS-R Estimated IQ	**Boston Naming Test ***(z score)*	**Verbal Fluency Test ***(z score)*	**Vigilance Test ***(errors)*	**WMS-R General Memory ***(z score)*	**WMS-R Verbal Memory ***(z score)*	**WMS-R Visual Memory ***(z score)*	**WMS-R Delayed Recall ***(z score)*
***Right MTLE***									
**1**	left	100	0.12	-0.63	0	1.23	1.53	-0.05	1.95
**2**	NA	97	-4.8	-0.63	0	0.4	0.68	-0.37	-0.24
**3**	left	86	-3.39	-0.19	0	-0.55	-0.54	-0.76	-1.02
**4**	left	100	-2.13	-0.85	1	0.46	-0.02	1.68	-0.45
**5**	left	115	-0.11	0.02	0	2.38	2.96	0.46	3.16
**6**	left	100	1.32	0.46	1	0.9	0.77	0.63	0.48
**7**	left	97	-0.58	-0.85	0	1.29	1.07	1.04	1.88
**8**	left	92	-1.11	NA	0	-0.04	0.07	-0.11	-1
**9**	left	NA	NA	NA	NA	NA	NA	NA	NA

									
***Left MTLE***									
**1**	left	89	-1.27	0.46	0	-0.04	-0.22	0.27	-0.03
**2**	left	88	-4.49	-0.85	0	-1.25	-1.12	-0.5	-0.24
**3**	left	80	-5.01	-1.02	1	-0.23	-0.22	-0.24	-1.3
**4**	NA	94	-1.26	-0.85	0	-0.76	-1.19	0.78	-0.77
**5**	left	94	-2.69	-0.19	0	-0.83	-1.55	0.59	-0.65
**6**	left	89	-1	1.34	0	-0.04	-0.09	0.01	-0.59
**7**	left	80	-7.97	-1.64	0	-1.83	-2.3	0.01	-1.73
**8**	left	NA	NA	NA	NA	NA	NA	NA	NA
**9**	left	86	-4.45	NA	0	-1.51	-1.12	-1.53	-3.15

									
**T-score**		**3.11**	1.96	0.03	0.61	**3.92**	**3.81**	1.03	**2.52**
**P-value**		**< 0.01**	0.07	0.98	0.55	**<0.005**	**<0.005**	0.32	**0.02**

RHA, right hippocampal atrophy; LHA, left hippocampal atrophy; NA, not available.

### Functional connectivity for intragroup comparisons

#### Seed in the left hippocampus (Figure 2)

**Figure 2:**
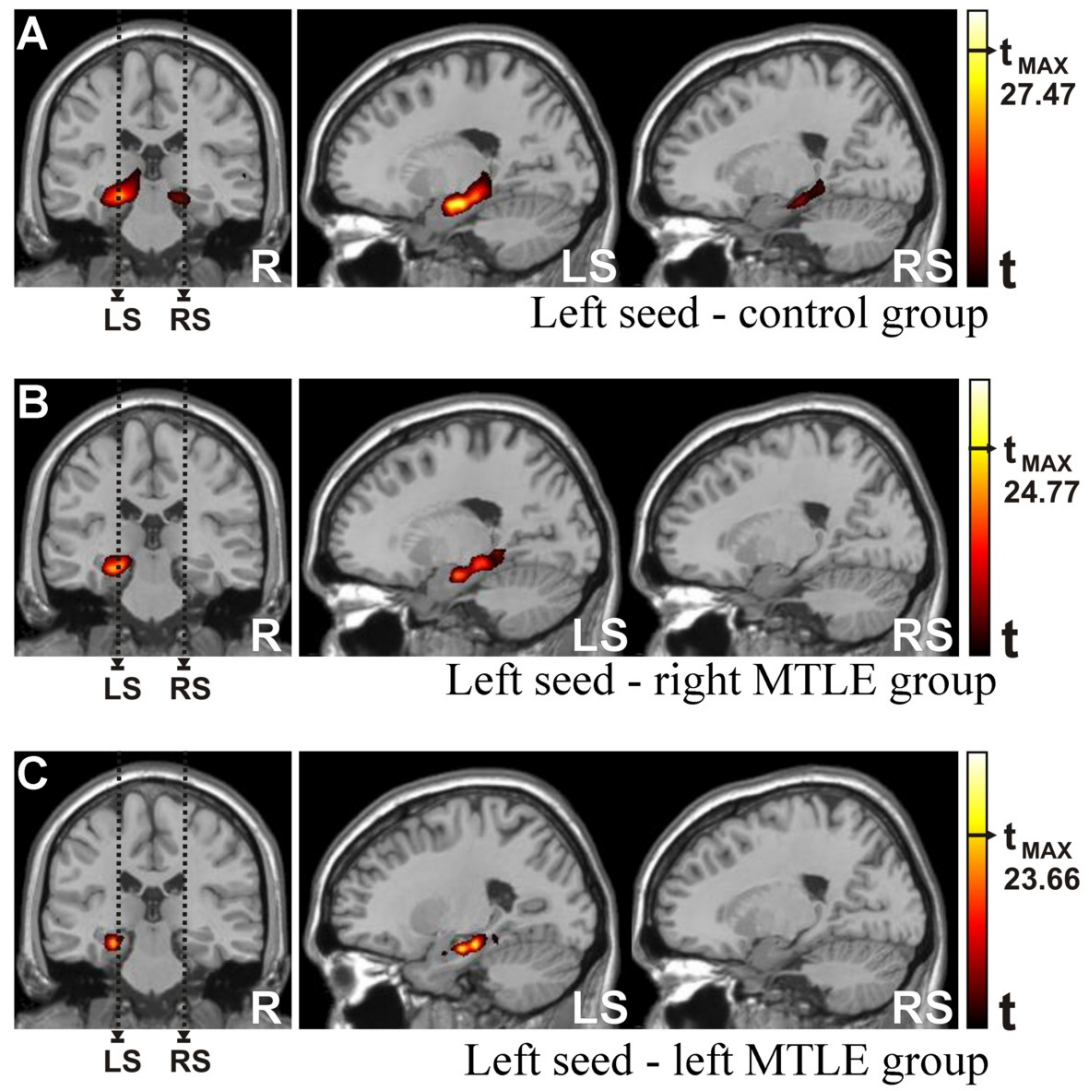
**Functional Connectivity Maps (intragroup comparisons)**. Left seed: a) Control group: Major cluster parameters (LS): T_max _= 27.47 at (-21,-13,-21) MNI coord. and 9813 voxels. Minor cluster parameters (RS): T_max _= 21.61 at (24,-21,-19) MNI coord. and 5271 voxels. b) Right MTLE patient group: Major cluster parameters (LS): T_max _= 24.77 at (-24,-16,-22) MNI coord. and 3315 voxels. c) Left MTLE patient group: Major cluster parameters (LS): T_max _= 23.66 at (-26,-26,-23) MNI coord. and 1599 voxels. Abbreviations - LS: left sagittal image, RS: right sagittal image. Statistical maps with t-scores higher than 15.

For the control group, we found a high level of functional connectivity with the left limbic lobe (cluster with 9813 voxels). The maximum t-score (t_max _= 27.47, p < 0.001) was located in the left parahippocampal gyrus at (x = -21, y = -13, z = -21) in MNI coordinates (Figure 2A - LS). We also detected a high correlation (T = 21.61, p < 0.001) with the right parahippocampal gyrus centered at (x = 24, y = -20, z = -19) with 5271 voxels in this cluster (Figure 2A - RS). The outcomes for the other two groups, corresponding to patients, were significantly different since there was no trace of left to right hippocampal functional connectivity above the threshold (T > 15, p < 0.001, k > 125 voxels) for either patient group (Figures 2B - RS and 2C - RS). Moreover, within the left mesial temporal lobe, the analysis revealed a functional connectivity map for the right MTLE group (Figure 2B) that was more widespread and had a stronger correlation than for the left MTLE group (Figure 2C). In quantitative terms, this means 3315 voxels and T = 24.77 for the right MTLE versus 1599 voxels and T = 23.66 for the left MTLE (p < 0.001). For right MTLE, the higher t-score was located at (x = -24, y = -16, z = -22) while for the left MTLE, at (x = -26, y = -26, z = -23).

#### Seed in the right hippocampus (Figure 3)

**Figure 3:**
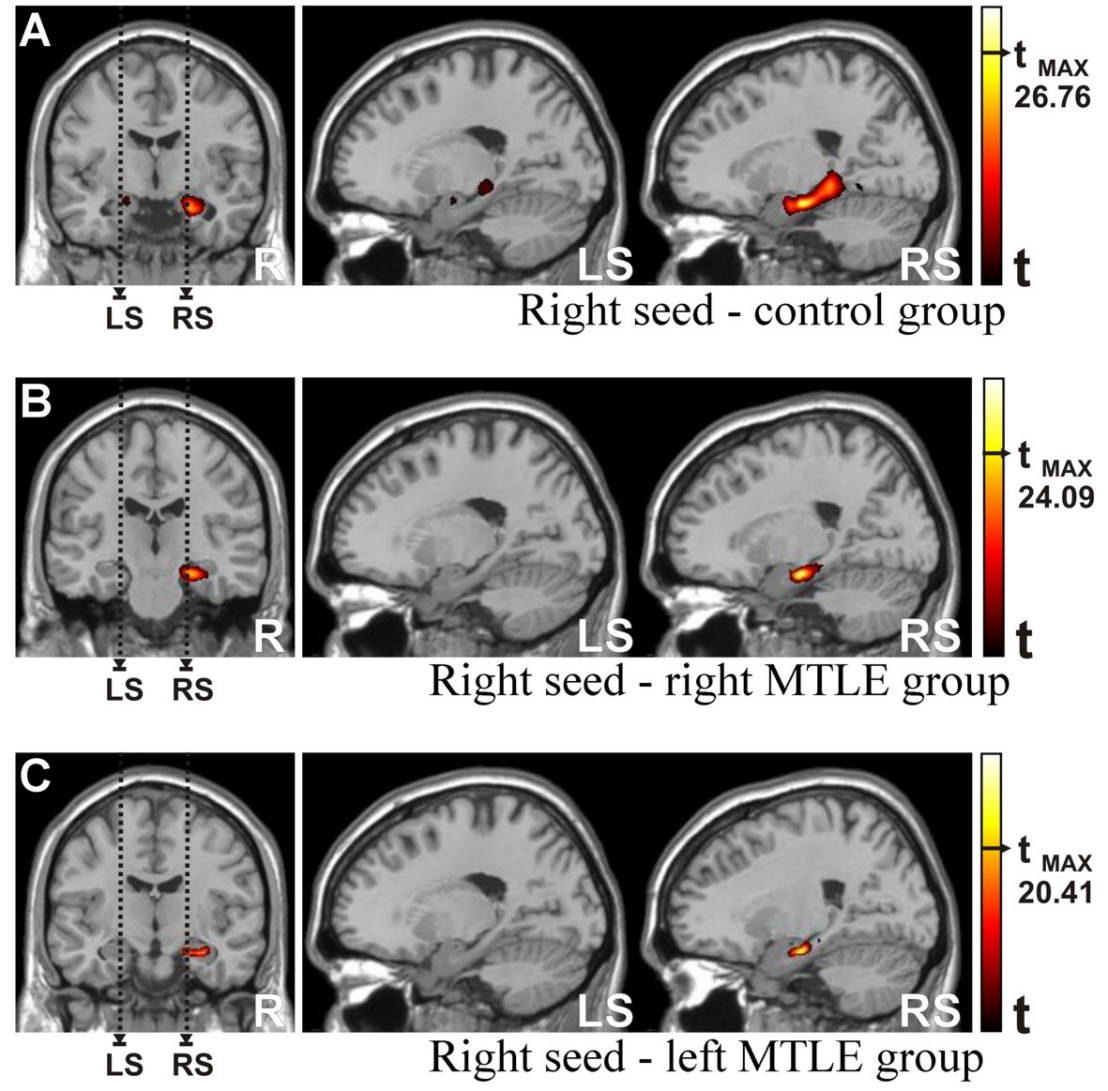
**Functional Connectivity Maps (intragroup comparisons)**. Right seed: a) Control group: Major cluster parameters (RS): T_max _= 26.76 at (28,-17,-20) MNI coord. and 6087 voxels. Minor cluster parameters (LS): T_max _= 23.79 at (-28,-19,-34) MNI coord. and 2466 voxels. b) Right MTLE patient group. Major cluster parameters (RS): T_max _= 24.09 at (20,-15,-22) MNI coord. and 1664 voxels. c) Left MTLE patient group. Major cluster parameters (RS): T_max _= 20.41 at (28,-16,-23) MNI coord. and 1179 voxels. Abbreviations - LS: left sagittal image, RS: right sagittal image. Statistical maps with t-scores higher than 15.

Analogously to what we obtained in the previous analysis, when we considered the seed in the right hippocampus, we found a strong correlation with voxels within other right mesial temporal lobe structures for the control group (T = 26.76, p < 0.001) centered at (x = 28, y = -17, z = -20), which is shown in Figure 3A. This correlation map was lower in intensity and smaller in cluster extent (6087 voxels) compared to the homologous situation described above (seed in the left hippocampus, Figure 2A). We were also able to detect a small region (2466 voxels) with significant correlation (T = 23.79, p < 0.001) in the left parahippocampal gyrus (x = -28, y = -19, z = -34), which can be seen in Figure 3A - LS.

The functional connectivity map showed a stronger correlation for the right MTLE group (Figure 3B) than for the left MTLE group (Figure 3C), with the maximum t-score of T = 24.09 and T = 20.41, respectively (p < 0.001), a pattern that is qualitatively similar to the situation observed for the left seed (previous section). The highest t-score for right MTLE was found at (x = 20, y = -15, z = -22) within a cluster of 1664 voxels and for the left MTLE, at (x = 28, y = -16, z = -23) within 1179 voxels as cluster size.

### Functional connectivity for intergroup comparisons

#### Seed in the left hippocampus (Figure 4)

**Figure 4:**
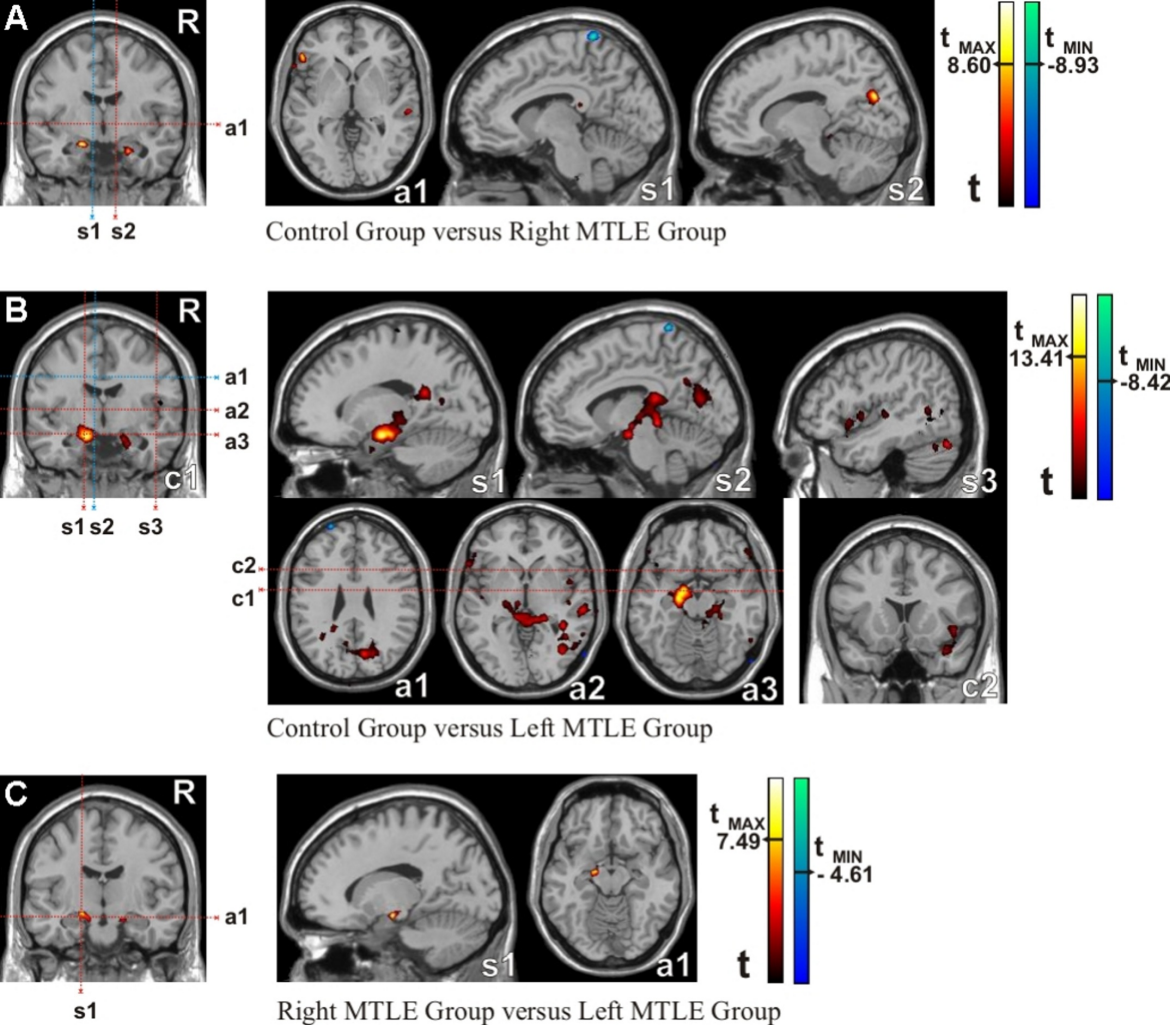
**Functional Connectivity Maps (intergroup comparisons)**. Left seed: a) Control group versus Patients with Right MTLE, b) Control group versus Patients with Left MTLE, c) Patients with Right MTLE versus Patients with Left MTLE. Abbreviations - s1: first sagittal image; s2: second sagittal image; s3: third sagittal image; a1: first axial image; a2: second axial image; a3: third axial image; c1: first coronal image; c2: second coronal image. Statistical maps with t-scores higher than 6.

Changes in functional connectivity between groups were detected using the two-sample t-test, pair by pair (see Methods). These analyses were performed in the following order: controls versus right MTLE, controls versus left MTLE and right versus left MTLE. The right MTLE group outcomes were similar to the controls, although the controls showed slightly stronger scores in a few areas, such as those illustrated by hot colors in Figure 4A. Basically, these regions comprised left (T = 8.60 at x = -19, y = -6, z = -18) and right (T = 7.78 at x = 22, y = -5, z = -29) parahippocampal gyri, triangular part of the left inferior frontal gyrus (T = 7.91 at x = -52, y = 34, z = 4) and cortex surrounding the calcarine fissure of the right hemisphere (T = 7.86 at x = 11, y = -73, z = 25).

The left MTLE group, on the other hand, had a large brain area with significant reduction of functional connectivity compared to controls (hot colors in Figure 4B). Fundamentally, this area extended bilaterally to large parts of the limbic lobes, especially in the mesial temporal lobe, (T = 13.41 centered at x = -17, y = -8, z = -21 on left hemisphere and T = 8.32 centered at x = 22, y = -4, z = -19 on right hemisphere) and subcortical gray nuclei (more significant on the left) such as the thalamus and amygdale. The right hemisphere also presented reduced functional connectivity scores in the left MTLE group compared to controls, mainly located in the lateral surface of the temporal lobe (T = 8.45 centered at x = 61, y = -26, z = 4).

The results shown in Figures 4A and 4B suggested that the left MTLE group had weaker connectivity than the right MTLE group, and that right MTLE patients presented connectivity patterns that were more similar to controls. This was confirmed by direct comparison between the left and right MTLE groups (Figure 4C), in which we detected some areas, in left (T = 7.49) and right (T = 6.43) mesial temporal lobe, of higher connectivity in favor of the right MTLE group (areas in hot colors) but none in favor of the left MTLE group.

The results presented in Figure 4 were obtained by imposing t-score higher than 6 and p < 0.001 for multiple comparisons.

#### Seed in the right hippocampus (Figure 5)

**Figure 5:**
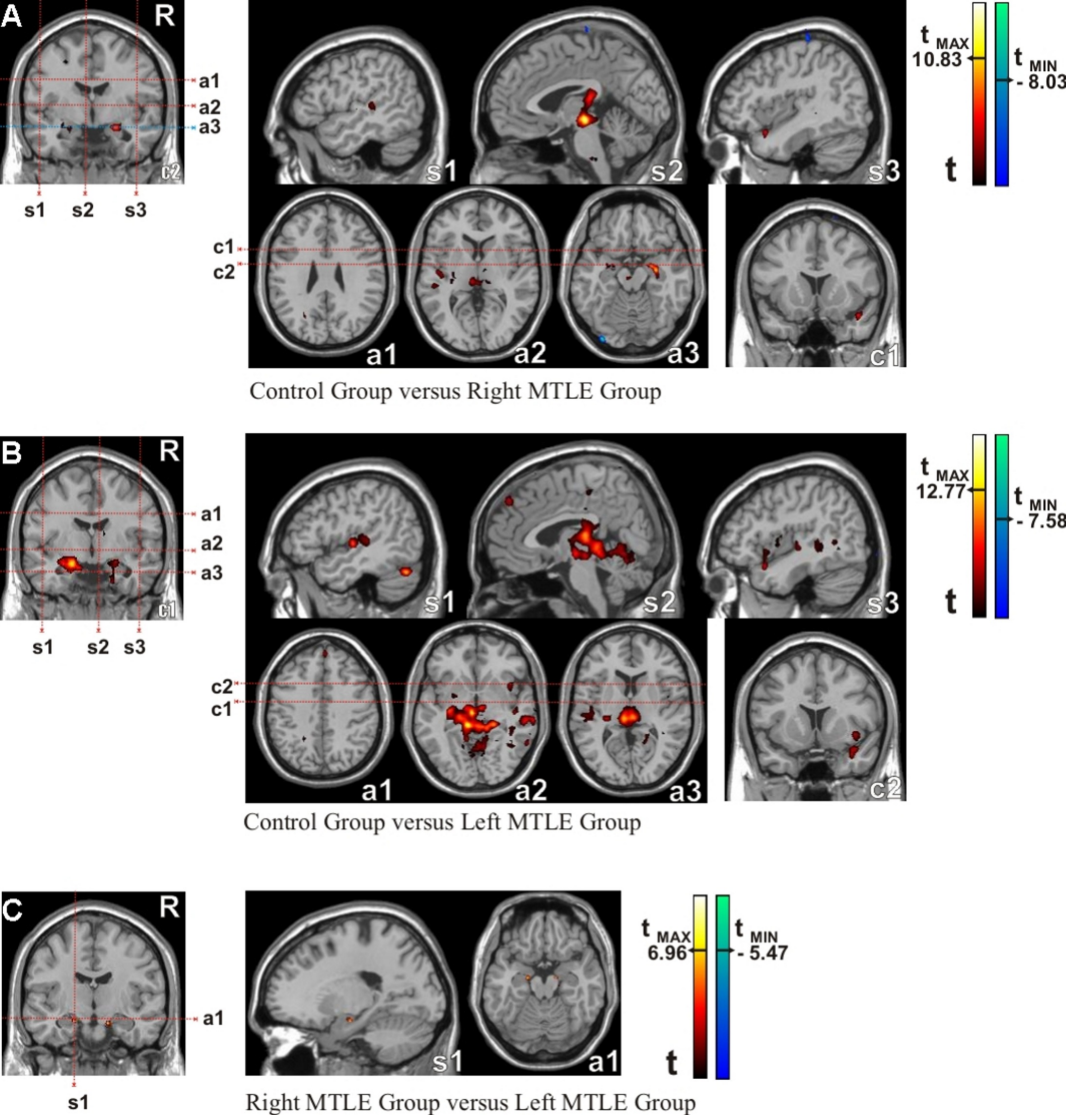
**Functional Connectivity Maps (Intergroup comparison)**. Right seed: a) Control group versus Patients with Right MTLE, b) Control group versus Patients with Left MTLE, c) Patients with Right MTLE versus Patients with Left MTLE. Abbreviations - s1: first sagittal image; s2: second sagittal image; s3: third sagittal image; a1: first axial image; a2: second axial image; a3: third axial image; c1: first coronal image; c2: second coronal image. Statistical maps with t-scores higher than 6.

Similar analyses were performed placing the seed in the right hippocampus. The control group presented more functional connectivity areas than the right and left MTLE groups (hot colors in Figures 5A and 5B, respectively). However, contrary to what was expected (since the seed was contralateral to the damaged hippocampus), the areas with more pronounced impaired functional connectivity were found in the left MTLE group. This finding was confirmed by direct comparison between the right and left MTLE groups (Figure 5C), by which we detected small areas of higher levels of functional connectivity (in red) in favor of the right MTLE group but none in favor of the left MTLE group. These small areas comprised basically the right (T = 6.95, x = 13, y = -10, z = -23) and the left (T = 6.64, x = -18, y = -10, z = -22) limbic structures.

### Analysis of the symmetries of the patterns of functional connectivity (Figure 6)

**Figure 6:**
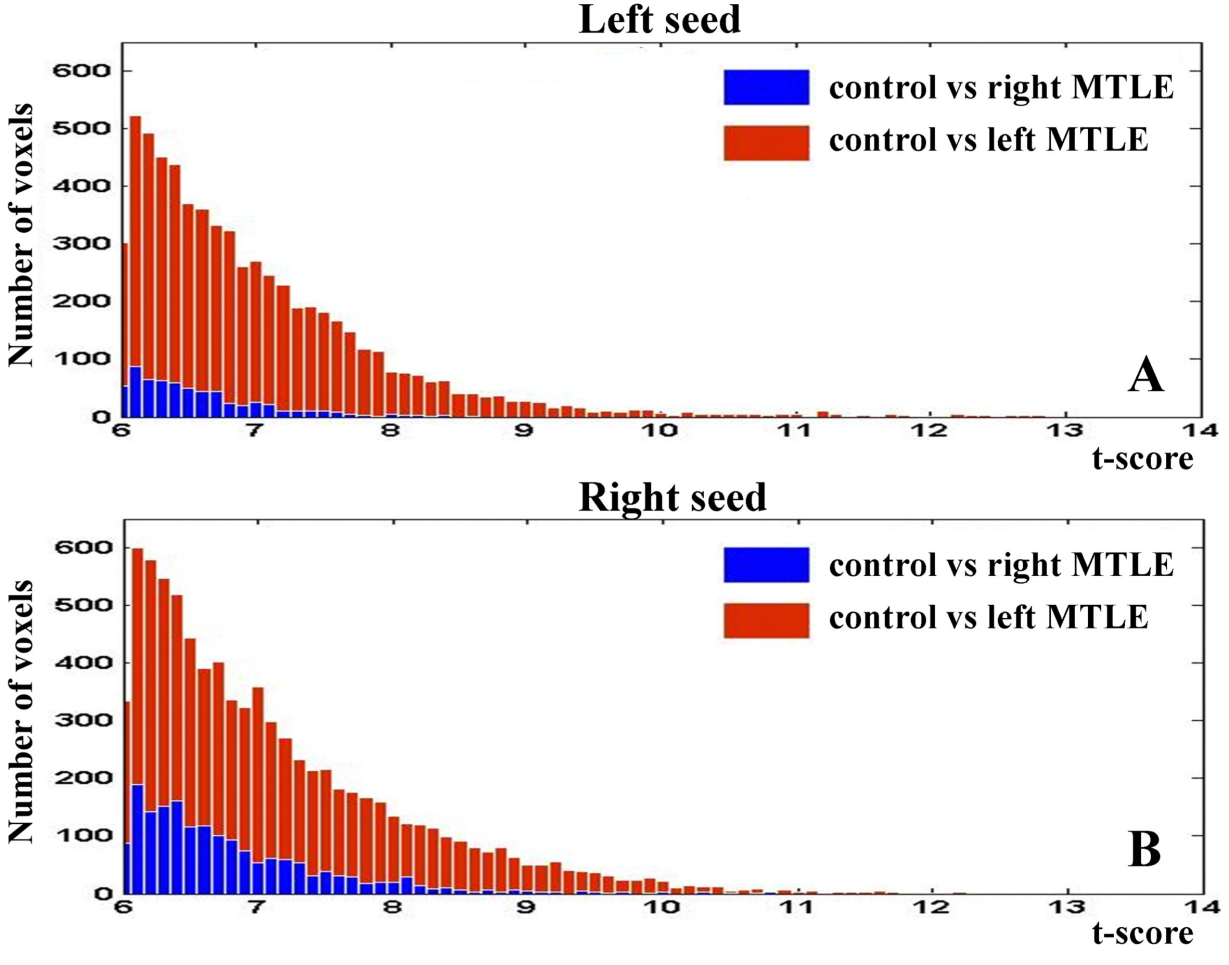
**Histogram of the Functional Connectivity Maps: intergroup comparisons**. A - Left seed: Blue line - control group versus right MTLE. The sum of the voxels was 630. Red line - control group versus left MTLE. The sum of the voxels was 6494. B - Right seed: Blue line - control group versus right MTLE. The sum of the voxels was 1765. Red line - control group versus left MTLE. The sum of the voxels was 8232.

In order to ascertain the differences between controls and patients, we computed the significant (T > 6, p < 0.001) voxels presented in Figures 4 and 5. These results are exhibited in the histogram of the Figure 6. In this figure, the blue bins represent the differences between control and right MTLE groups whereas the red bins indicate the differences between control and left MTLE groups. Thus, the area expressed by the sum of these bins reflects how different patients are to controls.

Concerning the left seed (Figure 6A), we counted 630 voxels in the blue bins (control versus right MTLE) and 6494 voxels in the red bins (control versus left MTLE). In respect to the right seed (Figure 6B), there are 1765 voxels in the blue bins (control versus right MTLE) and 8232 voxels in the red bins (control versus left MTLE).

Compared to controls, both patients' groups presented weaker patterns of functional connectivity, but the left MTLE group produced lower scores, even when the seed was placed in the right hippocampus.

## Discussion

The objective of this study was to explore differences of functional connectivity in patients with MTLE associated to unilateral (right or left) HS as compared to control subjects by means of resting state fMRI.

Patients and controls had similar age, educational level, handedness and hemispheric dominance for language. There was no difference in age of seizure onset, duration of epilepsy, seizure frequency and antiepileptic drugs used between the two patients' groups. They also presented similar degree of hippocampal atrophy (Figure 1 and Table 1).

Several studies have demonstrated that the left cerebral hemisphere is dominant for language for the majority of subjects [25,26] and damage in this hemisphere, such as HS, impairs more aspects of language than analogous damage in the contralateral hemisphere [6]. The basis of hemispheric language specialization deserves more studies, especially in terms of putative asymmetries in anatomical structures and/or functional patterns. Despite controversies, predictions of language lateralization can be accessed from gray matter probabilistic maps [27] as well as from white matter by means of diffusion tensor imaging [28]. Indeed, these works found high correlations between the density of gray and white matters and the dominant cerebral hemisphere for language. One may suppose that this asymmetric aspect is extendible to left/right hippocampal systems and try to evaluate this assumption by means of functional connectivity procedures [29]. Thus, we hypothesized that, in normal subjects with left hemisphere dominance for language, the network associated to the left hippocampus might have a higher level of functional connectivity than its analogous network on the right side, following the same pattern presented by previous structural studies [27,28]. To investigate this hypothesis, we excluded from this study subjects with atypical (bilateral or right-sided) language lateralization.

Taking into account that MTLE may cause cognitive deficits in human brain functions, such as memory and language, the sort of asymmetric pattern of functional connectivity discussed above might also occur in patients with this kind of epilepsy. In addition, decreases of the level of functional connectivity were observed ipsilateral to the damage in the MTL during the interictal period [30]. Although reduction of the levels of functional connectivity is not directly linked to the increase of functional damage, the progression of white and gray matter atrophies tends to be more intense in patients with left MTLE [31]. Indeed, asymmetrical extra-hippocampal gray matter loss related to hippocampal atrophy can encompass the ipsilateral and contralateral hemisphere, particularly the contralateral hippocampus, more pronounced in patients with left MTLE [32]. This asymmetrical pattern was also found in cryptogenic temporal lobe epilepsy, which has a distinct neuronal network, and the damages were, again, more widespread in patients with left-sided seizure focus [33].

By considering the groups of patients and their performance on neuropsychological tests, the group with left HS had lower IQ and worse performance on verbal memory, general memory and delayed recall than patients with right HS [34]. In fact, the correlations between the loss of gray matter and performance on a variety of neuropsychological domains also demonstrated higher scores for patients with left MTLE while no positive associations were found for patients with right MTLE [35]. These correlations were present at a "global level", and again, asymmetrical, suggesting involvement of a more pervasive network of these functions, which fits to the same pattern of functional connectivity that we found.

Vis-à-vis the convergence of the patterns of functional connectivity to the structural and functional evidences that came out with the previous works, which our study corroborates, we have looked for asymmetries in functional connectivity in three groups of subjects (patients with left MTLE, patients with right MTLE, and controls) as regards the functional network emulated by the left and right hippocampi during resting state in subjects with left hemisphere dominant for language.

The functional connectivity from the left to the right hippocampus and vice-versa was seen only in the control group (Figures 2A-RS and 3A-LS) as small spots of positive correlations contralateral to the seed location. Similar outcomes were not found in patients' group, neither for the right MTLE group (Figures 2B-RS and 3B-LS) nor for the left MTLE group (Figures 2C-RS and 3C-LS). These findings show that the patterns of functional connectivity of MTLE patients are disrupted.

Still concerning to the intragroup comparison, the major cluster and the maximum t-score were found in areas of the MTL ipsilateral to the seed. For the three groups, the patterns of functional connectivity were higher on left hemisphere. This asymmetry fits precisely to those works that related language lateralization prediction from brain structures imaging [27,28], but here, the basis of the asymmetry is directly related to the hippocampal basal functional network.

Besides the patterns of functional connectivity were higher for the left seed, there was a hierarchy among the three groups. Controls presented higher correlations than the right MTLE group, which exhibited higher levels than the left MTLE group (Figures 2A, 2B and 2C, respectively). Identical order was found for the right seed which is illustrated by Figures 3A, 3B and 3C respectively. Since the hierarchy of these groups concerned to the homogeneity of the seeds, the patients with left MTLE presented more variability than the other two groups for both hippocampi. By the same rule, the right MTLE had more variability than control subject for hippocampi time series. For these patients with unilateral MTLE, these findings suggests that the functions which depend on hippocampi may be damaged, displaced or rearranged due to the pathological condition [36] or to the compensatory mechanisms [30].

In general, controls exhibited higher patterns of functional connectivity than patients, but the t-scores and the cluster sizes of these intergroup comparisons had distinct values in accordance of two elements: the used seed and the side of HS.

For the seed ipsilateral to the HS, both patient groups presented important reductions of functional connectivity in several brain regions. The Figures 4B and 5A illustrate these regions for the left and the right MTLE groups respectively. These findings could be explained by the ipsilateral hippocampal atrophy associated to the epileptogenic dysfunction, which may reduce the functional connectivity [30]. This explanation, based on a structural hypothesis, indicates an intrinsic relationship between the anatomical injury and the disrupted basal functional connectivity [37].

Besides the fact that a decrease of the functional connectivity patterns encompasses several brain regions for patients, the left MTLE group presented more of these regions with reduction than the right MTLE group. Indeed, these findings are illustrated by two aspects. First, the maximum t-scores were 13.41 for controls versus left MTLE group (Figure 4B) and 10.83 for controls versus right MTLE group (Figure 5A). Second, the amount of significant voxels was higher for the comparisons between controls and left MTLE group as shown in Figure 6. These intergroup comparisons make evident the reduction of the functional connectivity patterns in MTLE patients and suggest that left HS is associated to higher decrease of functional connectivity than right HS.

For the seed contralateral to HS, we also found a reduced functional connectivity in the left MTLE group as compared to the right MTLE group, as demonstrated by t-scores (Figure 4) and number of significant voxels shown in Figure 6. These outcomes were unexpected since we evaluated the "healthier" hippocampi in each group. This astonishing result strongly suggests the existence of distinct functional basal brain processes associated to the left or to the right HS, since left MTLE group holds off controls where right MTLE group approaches to then.

In order to confirm the hierarchy of the three groups, which were firstly suggested by intragroup and then reinforced by intergroup comparisons, we searched the brain regions that were reduced in the left MTLE when compared to right MTLE. By using the left seed, we found two small clusters in both hippocampi (Figure 4C). The highest t-score was 7.49. By using the right seed, we found two similar clusters (Figure 5C) but the highest t-score was 6.96. Taken together, these findings pose stronger evidences that, in the context of a similar degree of hippocampal atrophy, left HS is associated to more reduction of the hippocampal functional connectivity than the right HS.

The unilateral HS was correlated with abnormal patterns of connectivity in other parts of the brain, not only ipsilateral to the hippocampal atrophy [38]. Indeed, the patterns of disrupted connectivity diverged in the MTLE patients' groups, being even lower in the left MTLE group. This result could be explained by more structural damage (i.e. more intense hippocampal atrophy) in the left MTLE. However, this was not the case here, since both patients' groups had similar degree of hippocampal atrophy confirmed by MRI volumetric analysis. Another possibility could be differences in the regional distribution of atrophy within hippocampi, with more pronounced damage in specific parts of each hippocampus, affecting different fiber projections to and from the hippocampal system, thus, causing distinct functional disruptions [39,40]. Secondly, for patients with left hemisphere dominance for language, left MTLE may cause more functional connectivity damage in the contralateral hippocampus than right MTLE does, because a disruption in the non-dominant hemisphere (the right one in this case) paradoxically may accelerate verbal processes [41], and may not disturb the basal brain organization in a critical way. Thirdly, in control subjects with left hemisphere dominance for language, the influence of the left hippocampus on the right hippocampus could be more significant than the reverse influence during the resting state, probably due to the hierarchy of brain organization. This is supported by the fact that the seed in the left hippocampus had a higher correlation with the right hippocampus than vice versa (Figures 2A and 3A). These findings indicate that the time series of the left hippocampus is more homogeneous than the time series of the right hippocampus for the control group. In any case, these assumptions are based on evidence from functional MRI and require further experiments for confirmation, such as perfusion, tensor imaging, etc. However, these results are robust enough to demonstrate that although no MRI structural damage could be seen in the hippocampus contralateral to the seizure focus, significant functional brain plasticity might have occurred in these patients. This plasticity differed between left and right MTLE, suggesting that we should use distinct ways of addressing the functional organization of the brain in patients with MTLE.

In addition to the above caveats, it should be noted that our modest sample size represents an important limitation although a large number or runs were performed for each subject. The issue of the statistical power might be more relevant for patients, especially for left MTLE group which exhibited higher levels of variability than the other groups. A further reservation should be stated about the filters applied during the pre-processing period. As functional connectivity correlates two time series, there might exist functional connectivity between signals oscillating out of the band pass that we used. These interactions were practically impossible to remove if they were synchronized to the physiological noise and, to prevent any contamination; we took the risk of excluding genuine connectivity by filtering frequencies out of the band pass. In addition to the fact that this option is quite conservative, we applied the same steps for all groups.

Another important limitation is related to the mask used. Although, the automated anatomical labeling (AAL) atlas [42] was made for MNI coordinates, it was segmented concerning only one subject. Moreover, this segmentation was made on a structural image, different from the EPI used for our functional data. To reduce the bias added by the masking period, we applied the same mask (left and right hippocampi masks) for all subjects.

## Conclusions

We demonstrated that the method of determining functional connectivity by means of resting state fMRI was sensitive enough to detect differences between patients with right and left MTLE versus controls in the organization of inter-relationships between brain regions [43]. This study also showed a dysfunction in the interconnections of the epileptogenic hippocampus in patients with MTLE, which may be related to anatomical (atrophy) or functional (interictal epileptiform spikes) abnormalities, or both [44]. Indeed, left HS causes more reduction of the functional connectivity than right HS in subjects with left hemisphere dominance for language, and may be related to common dysfunction suffered by patients with left MTLE. These evidences reinforce a distinct role followed by each hippocampus in the brain organization and suggest that we should use distinct approaches to deal with patients with left and right MTLE.

## Methods

### Subjects

We studied 27 subjects: 9 control subjects (5 men, mean age 33 ± 9 years); 9 patients with chronic refractory right-sided MTLE (4 men, mean age 39 ± 6 years) - right MTLE group; and 9 patients with chronic refractory left-sided MTLE (1 man, mean age 35 ± 9 years) - left MTLE group. There were no significant differences in age between controls versus patients with right MTLE (F = 3.16; p = 0.10), controls versus patients with left MTLE (F = 0.34; p = 0.57) nor between patient groups (F = 1.05; p = 0.32). Moreover, patient groups showed no statistically significant differences in terms of the age at seizure onset (F = 0.62; p = 0.25), anti-epileptic drugs used (F = 1.29; p = 0.28) and seizures frequency (F = 0.001; p = 0.99). All subjects had left hemisphere dominance for language according to the Dichotic Listening Test, and all were right-handed according to the Edinburgh Handedness Inventory [45]. All subjects participating in this study gave their informed consent in accordance with the Research Ethics Committee of the University of Campinas - UNICAMP.

Patients were diagnosed based on their clinical history and physical examination results, as well as on EEG and MRI investigations. The diagnoses of epilepsy were classified according to criteria from the commission on classification and terminology of the International League Against Epilepsy (1989). Seizures were lateralized according to the medical history, a comprehensive neurological examination, interictal EEG and video-EEG monitoring for seizure recording. Consistent lateralization was seen in at least six interictal EEGs and two seizures recorded during video-EEG monitoring. Visual analyses of structural MRIs demonstrated unilateral hippocampal atrophy [3] and other signs of HS. All patients were considered to have drug-refractory MTLE with unilateral seizure onset ipsilateral to the hippocampal atrophy.

### Data acquisition

#### MRI and fMRI data

All images were acquired on a 2T MRI scanner (Elscint Prestige, Haifa, Israel). Structural high-resolution T1-weighted gradient echo sagittal images were acquired using a gradient-echo sequence with TR = 22 ms; TE = 9 ms; flip angle = 35°; thickness = 1 mm; matrix = 256 × 256 and isotropic voxels (1 × 1 × 1 mm^3 ^voxels), for multiplanar reconstruction and hippocampal volumetric measurements. As part of our MRI epilepsy protocol, all patients had axial images: T1-weighted and FLAIR (Fluid attenuated inversion recovery); and coronal images: T1-inversion recovery, T2-weighted and FLAIR.

For functional MRI, T2*-weighted axial echo planar images (EPI) were acquired in an interleaved mode by using a gradient-echo sequence with TR = 2000 ms; TE = 45 ms; flip angle = 90°; thickness = 6 mm; matrix = 128 × 72; 3 × 3 × 6 mm^3 ^voxels and 20 slices per volume. For resting state scans, subjects were instructed to rest and not to think of anything in particular. During these acquisitions, sensory stimulation was limited to the noise of the scanner. To reduce it, all subjects wore earplugs. Moreover, all subjects had their head movements restricted by a soft velcro strap. For each subject, we acquired 10 runs in the resting state condition, each run lasting 6 min and 10 seconds.

#### Neuropsychological evaluation

Both patient groups were submitted to a comprehensive neuropsychological evaluation, which included: (1) vocabulary and block design subtests of the Wechsler Adult Intelligence Scale--Revised (WAIS-R) to estimate IQ; (2) the Edinburgh Handedness Inventory and Dichotic Listening Test to determine hemispheric dominance for language and, by inference, to lateralize verbal and visual memories; (3) the Logical Memory and Verbal Paired Associates of the Wechsler Memory Scale--Revised (WMS-R) to investigate verbal memory; and (4) the Figural Memory, Visual Reproduction, and Visual Paired Associates of the WMS-R to investigate visual memory. They were also submitted to tests for language (Verbal Fluency Test and Boston Naming Test/BNT), and attention (Strub and Black Vigilance Test) [45-51].

### Data analysis

MRI, EEG and clinical findings were analyzed by our group of experts on investigations for epilepsy surgery and all patients were confirmed as having unilateral MTLE, including unilateral hippocampal atrophy on MRI visual analyses. Manual volumetric measurements of hippocampi were performed on 3D-gradient echo images using the Display-3 D and Volume Program [52] according to our previously published protocol [53]. For each subject, a quantitative asymmetric index (QAI) was calculated by subtracting the quotient between the volume of the smaller (VSH) and the bigger (VBH) hippocampi from unit (QAI = 1 - Q, where Q = VSH/VBH).

EPI images were reconstructed from k-space by using homemade MATLAB [54] routines and ghost artifacts were reduced by applying the algorithm proposed by Buonocore and Gao [55]. Next, these images were converted to the ANALYZE file format using the MRIcro software [52,56,57]. The first five images were discarded to guarantee stable baseline data at the beginning of each run. After that, we applied the following four steps within SPM5 [58]: 1) temporal shifting with slice timing correction for interleaved acquisition; 2) motion correction for each subject; 3) normalization to match the overall size and shape of the data; and 4) smoothing of all EPI images with a 6 mm FWHM Gaussian kernel. Next, all smoothed images were filtered with high-pass (f >0.01 Hz) and low-pass (f<0.08 Hz) temporal filters to reduce the effect of low-frequency drifts and high-frequency noise [59] by using the Analysis of Functional NeuroImages (AFNI) software [60,61].

### Seed generation

We defined two anatomical volumes of interest (AVOI) corresponding to the left and right hippocampi by masking these brain structures with the automated anatomical labeling (AAL) atlas [42], provided by the MRIcron software [57]. Then, we extracted the time series from the voxels within these AVOIs for all subjects, and averaged these time series in order to obtain two seeds (left seed and right seed) to be used as reference in the subsequent analysis.

### Functional connectivity analysis

The time series of each seed (corresponding to the left and right hippocampi) were correlated with all time series over the brain with a threshold at *p *< 0.0001 (uncorrected). All negative correlations were removed from subsequent analysis steps. The correlation coefficients (r) were converted to t-values according to the standard transformation [62]:

where n is the number of time points. Fisher's z-transform was then applied to the t-values in order to normalize the Student's t-parameter distribution [63]. These z-values were entered into a random-effect one-sample t-test for each subject [64], with the purpose of determining intragroup functional connectivity maps. The z-values were also entered into a second-level random-effect analysis to determine brain areas with significant functional connectivity across groups. We performed these two-sample t-tests as a means to detect differences in controls versus left MTLE patients and controls versus right MTLE patients.

All intra and intergroup statistical analyses had a threshold at *p *< 0.001 (corrected for multiple comparisons) at the voxel level and the volume of the cluster surrounded by each mesial temporal lobe were computed by eliminating the clusters with less than 1 cm^3 ^(125 voxels).

## Authors' contributions

FRSP conceived of the study, carried out the functional connectivity studies, participated in the image acquisition, analyzed the data images, performed statistical analysis and drafted the manuscript. AA participated in the image acquisition and carried out the neuropsychological studies. MSS participated in the image acquisition and reconstructed the images. TP participated in the image acquisition and reconstruction, and performed hippocampal volumetry. EB participated in the image acquisition and selected the patients. JMR participated in the image acquisition and reconstruction. HFBO participated in the image acquisition and reconstruction. GC participated in study design, developed computational routines and drafted the manuscript. RJMC participated in study design and drafted the manuscript. BPD participated in study design and in the data interpretation. FC conceived of the study and participated in its design and coordination, drafting and revision, as well as funding support. All authors read and approved the final manuscript.
